# Real-world prescription patterns for reproductive assistance patients in China: A trend analysis from 2016 to 2020

**DOI:** 10.3389/fphar.2022.1021150

**Published:** 2022-11-17

**Authors:** Jing Jin, Jialei Zhu, Jing Tang

**Affiliations:** Department of Pharmacy, The Obstetrics and Gynecology Hospital of Fudan University, Shanghai, China

**Keywords:** China, cross-sectional analysis, infertility, drug treatment model, real world

## Abstract

**Background:** Pharmacotherapy is one of the primary treatments for patients with Assisted reproductive technology (ART). Despite the publication of various research on ART treatment, there is no clear conclusion regarding the choice of drug treatment in China. Our research intends to examine the trend of widely prescribed medications for ART patients in China. For instance, the study examines the logic of drug indications, usage, and dose in patient prescriptions.

**Methods:** We did a cross-sectional study of the data from the hospital prescription analysis cooperation project supervised by the China Medical Association. The information is extracted from the prescriptions of reproductive assistance outpatients from January 2016 to December 2020. We used the U.S. Food and Drug Administration (FDA) classification to quantify the frequency of drug use and the categories of drugs. We manually extract the information of patients who require ART treatment, divide the patients into various age groups and geographies, followed by study the indications, utilization, and rationale of the most important therapeutic medications.

**Results:** Among the 225225 patients included in this study, Guangzhou (47.83%), Shanghai (19.84%), and Zhengzhou (9.36%) were the top three cities. In the past 5 years, the average age was 32.99, and 60.38% of women were between the age of 25 and 34. The main therapeutic medicines taken by each patient, primarily hormone therapies, were tallied. Eleven types of primary therapeutic medicines were employed. Different progesterone preparations (47472, 21.08%), chorionic gonadotrophin gondotrophin for injection (38932, 17.29%), dydrogesterone tables (33591, 14.91%), and triptorelin for injection (26959, 11.97%) rounded out the top five. According to the data on outpatient medications in major cities in China, the variety and proportion of injections are the highest, including the most frequent types of ovulation induction and urotropia, as well as triptorelin and progesterone. Even though the total dosage of urotropin was the highest in 5 years, it showed a declining trend. The dosages of progesterone and didroxyprogesterone increased, with progesterone showing the most rapid increase. The top five most expensive prescription medications are triptorelin, urotropin, progesterone, didroxyprogesterone, and leuprorelin, in that order. Goserelin, leuprorelin, triptorelin, growth hormone, and didroxyprogesterone are among the top five most expensive medications per capita.

**Conclusion:** The average age of patients has not increased considerably over the past 5 years. However, the opportunity cost of childbirth for women has increased, which has significantly enhanced their willingness for childbearing intentions. The medication selection is reasonable overall. In this study, the recommended dosages of first-line medicines (urotropin and chorionic gonadotropin) are likewise high. In contrast, the dosage of oral first-line treatment for ovarian stimulation in unexplained infertility is modest, and the dosage of progesterone is steadily increasing. In addition, the price of certain medicines is high, which will increase the patients’ financial burden. Future research will focus on enhancing the degree of rational drug use among outpatients and realizing the economical, safe, and effective use of pharmaceuticals to lessen the economic burden of patients.

## Introduction

Infertility is defined as failure to achieve clinical pregnancy 1 year after regular unprotected intercourse (Zegers-Hochschild et al., 2017). In 2016, China recorded more than 40 million cases of infertility, representing 12.5% of the reproductive age population (Liu et al., 2019). With the liberalization of China’s fertility policy, many older women exhibited a strong desire for fertility, and the number of patients obtaining assisted reproductive technology for pregnancy rose annually (Yang and Zhang, 2021). It is a crucial component of the International Conference on Population Development (ICPD) declaration from almost 25 years ago (Polis et al., 2017).

The most recent global evaluation commissioned by the World Health Organization (WHO) in 2010 found that up to 48 million couples worldwide has suffered from infertility, with low-and middle-income countries (LMIC) bearing half of the global burden (Gerrits et al., 2017). A paucity of data and high fertility rates in many LMICs conceals the underlying burden of infertility, which is likely an underestimate (Mascarenhas et al., 2012). From 1980 to 1999, the cohort revealed an increase in US women diagnosed with primary infertility. From 1980–1985 to 1995–1999, ART rates increased from 1.8% to 26.0%. Between 1980–1985 and 1995–1999, the incidence of primary infertility increased from 14 per 10,000 person years to 20 per 10,000 person years.

The most common reasons for primary infertility were ovulatory dysfunction and unexplained infertility, whereas clomiphene has been prescribed most widely among fertility drug (Sadecki et al., 2022). ART development and administration can considerably raise the clinical pregnancy rate. Ovulation induction therapy is one of the most essential components of ART. Clomiphene (CC), aromatase inhibitors, gonadotropins (Gn), and gonadotropin releasing hormone analogues (GnRHa), including agonists (GnRH-a) and antagonists, are the drugs most frequently used to induce ovulation (GnRH-A). GnRH-A use has also gradually increased in recent years (Jie et al., 2015). However, there is no authoritative literature report on the specific drug treatment methods. How to employ ovulation-inducing drugs and develop standard monitoring and evaluation indicators has become an increasingly important clinical issue for reproductive physicians (Hajishafiha et al., 2013; Filicori and Cognigni, 2018; Practice Committees of the American Society for Reproductive Medicine and Society for Reproductive Endocrinology and Infertility., 2020; Insogna and Ginsburg, 2021; Nesbit et al., 2022).

At present, there are limited studies on infertility therapy for patients undergoing ART in China, and there is no unified conclusion on the choice of drug therapy. Therefore, our study focuses on the pharmacological use for infertility therapy patients in china over the past 5 years to fill these gaps.

## Materials and methods

We used data from the China Medical Association’s Hospital Prescription Analysis Cooperation Project for rational use of drugs, which was a national database designed for monitoring rationality of drug use. Approximately 120 hospitals that uploaded diagnosis and prescription information were included from provincial-level regions of China from 2016 to 2020. The centres are general or specialized governmental hospitals in Beijing, Chengdu, Guangzhou, Harbin, Hangzhou, Shanghai, Shenyang, Tianjin, and Zhengzhou. North China, East China, South China, Central China, and other regions are represented.

We manually extracted the information of patients who require ART treatment, divided the patients into various age groups and geographies, and studied the indications, utilization, and rationale of the most essential therapeutic medications. The following information was collected: visiting time, city, hospital code, method of medical administration, unit cost, administration frequency, single dose, dosage, age, and initial diagnosis.

This study extracted the outpatient prescription data of women diagnosed with unexplained primary and secondary infertility from 1 January 2016 to 31 December 2020. It also included combined diagnosis of infertility caused by other reasons, such as “infertility caused by non ovulation,” “infertility caused by fallopian tube,” “habitual abortion,” “chronic salpingitis,” “urethritis,” “polycystic ovary syndrome,” “hyperandrogenemia,” “missed abortion,” “pelvic inflammation,” “immune deficiency,” “ovarian cyst,” “insulin resistance,” “cervicitis,” “vaginitis” “Fungal vaginitis,” “mycoplasma infection,” “intrauterine adhesion,” “ovarian dysfunction,” “adenomyosis,” “hypothyroidism,” “endometritis,” “endometriosis,” etc. The drugs of the project are limited to western medicine, and some traditional Chinese medicine prescriptions are excluded.

For further analysis, we divided the patients into different age groups and geographies, selected the most critical therapeutic drugs, and further analyzed them according to drug selection, administration route, drug dosage and rationality (Hajishafiha et al., 2013). According to the pharmacological classification of main therapeutic drugs, the sales amount of drugs consumed in the past 2 years is counted, and the proportion of sales amount in the total sales of Keane is calculated and sorted, as well as according to the World Health Organization (WHO) and the defined daily dose (DDD) system. The DDD value is determined referring to the instructions for clinical medication (2010 Edition) (Sun and Wu, 2015) and newly compiled Pharmacology (17th Edition) (Chinese Pharmacopoeia Commission. Pharmacopoeia of the people’s Republic of China, 2010), combined with the clinical medication and drug instructions. For rationality analysis, we evaluated the hormone prescription’s complete frequency and single dose information according to the scheme recommended by the drug label and the latest Chinese guidelines.

### Statistical analysis

Statistical analysis was conducted using Excel 2013 and SPSS software (version 25; SPSSInc., Chicago, IL, United States). Continuous variables are shown as mean ± standard deviation. Categorical variables are presented as numbers and percentages. Demographic and prescription information was grouped into counts.

## Results

### Demographic characteristics of patients

According to the inclusion and exclusion criteria standard, we included the prescription data of 225225 patients in the project and analyzed relevant information, such as geography, reimbursement method, expenses, etc. The top three cities where the patients from are Guangzhou (47.83%), Shanghai (19.84%) and Zhengzhou (9.36%). The average age in 5 years is 32.99. The 25-to-34-year-old age range accounts for 60.38% of the entire age range, the largest proportion. The demographic characteristics of specific patients are detailed in Table 1.

**TABLE 1:** Demographic characteristics of the patients (*n* = 225225).

Year	2016	2017	2018	2019	2020	Total
Region, n (%)						
Beijing	3,120 (1.39%)	3,305 (1.47%)	2,915 (1.29%)	3,042 (1.35%)	1,693 (0.75%)	14075 (6.25%)
Chengdu	2,375 (1.05%)	3,934 (1.75%)	3,431 (1.52%)	3,977 (1.77%)	2,516 (1.12%)	16233 (7.21%)
Guangzhou	20154 (8.95%)	25343 (11.25%)	21116 (9.38%)	24376 (10.82%)	16733 (7.43%)	107722 (47.83%)
Haerbin	1,504 (0.67%)	1,444 (0.64%)	1,537 (0.68%)	1708 (0.76%)	625 (0.28%)	6,818 (3.03%)
Hangzhou	761 (0.34%)	1,149 (0.51%)	852 (0.38%)	1,382 (0.61%)	2,110 (0.94%)	6,254 (2.78%)
Shanghai	11646 (5.17%)	8,203 (3.64%)	9,904 (4.40%)	8,939 (3.97%)	5,987 (2.66%)	44679 (19.84%)
Shenyang	8 (0.00%)	3 (0.00%)	5 (0.00%)	6 (0.00%)	3 (0.00%)	25 (0.01%)
Tianjin	432 (0.19%)	2,948 (1.31%)	705 (0.31%)	2024 (0.90%)	2,224 (0.99%)	8,333 (3.70%)
Zhengzhou	4,831 (2.14%)	3,874 (1.72%)	4,511 (2.00%)	3,826 (1.70%)	4,044 (1.80%)	21086 (9.36%)
Total	44831 (19.90%)	44976 (19.97%)	49280 (21.88%)	50203 (22.29%)	35935 (15.96%)	225225 (100%)
Drug costs	9413965.68 (18.28%)	9828855.14 (19.08%)	11255979.89 (21.85%)	12392707.38 (24.06%)	8614503.64 (16.73%)	51506011.73 (100%)
Age, n (%)						
18–24	1746 (0.78%)	1776 (0.79%)	1770 (0.79%)	1963 (0.87%)	1,230 (0.55%)	8,485 (3.77%)
25–34	26575 (11.80%)	30542 (13.56%)	26419 (11.73%)	29761 (13.21%)	22683 (10.07%)	135980 (60.38%)
35–44	15125 (6.72%)	16643 (7.39%)	15465 (6.87%)	16304 (7.24%)	11354 (5.04%)	74891 (33.25%)
45–50	1,385 (0.61%)	1,242 (0.55%)	1,322 (0.59%)	1,252 (0.56%)	668 (0.30%)	5,869 (2.61%)
Average	33.08	33.10	32.93	33.04	32.79	32.99

### Type of medication used by the patient

We counted the main therapeutic drugs taken by each patient and discovered that they were mainly hormone drugs. There are 11 kinds of primary therapeutic medicines employed. The top five are urotropin for injection (47932, 21.28%), progesterone soft capsules (47472, 21.08%), chorionic gonadotropin for injection (38932, 17.29%), dydrogesterone tables (33591, 14.91%) and triptorelin for injection (26959, 11.97%). See Table 2 for details.

**TABLE 2:** Main therapeutic drugs of the patients (*n* = 225225).

Drug classification	Main therapeutic drugs	2016	2017	2018	2019	2020	Average	Mean age
Gonadotropin	Urotropin for injection	12678 (28.28%)	10439 (23.21%)	9,598 (19.48%)	9,045 (18.02%)	6,172 (17.18%)	47932 (21.28%)	32.93 ± 5.59
Progesterone drugs	Progesterone	8,036 (17.93%)	9,564 (21.26%)	11206 (22.74%)	10757 (21.43%)	7,909 (22.01%)	47472 (21.08%)	32.63 ± 5.16
Gonadotropin	Chorinonic gonadotrophin for injection	7,968 (17.77%)	7,362 (16.37%)	8,790 (17.84%)	8,845 (17.62%)	5,967 (16.60%)	38932 (17.29%)	33.49 ± 5.53
Progesterone drugs	Dydrogesterone tablets	5,673 (12.65%)	6,072 (13.50%)	7,121 (14.45%)	8,403 (16.74%)	6,322 (17.59%)	33591 (14.91%)	32.58 ± 5.12
Gonadotropin releasing hormone analogue	Triptorelin for injection	5,083 (11.34%)	5,445 (12.11%)	5,951 (12.08%)	6,476 (12.90%)	4,004 (11.14%)	26959 (11.97%)	33.17 ± 5.29
Aromatase inhibitor	Letrozole tablets	1821 (4.06%)	2,111 (4.69%)	2,345 (4.76%)	2,636 (5.25%)	1968 (5.48%)	10881 (4.83%)	31.94 ± 5.25
Anti estrogen drugs	Clomifene citrate tablets	1,687 (3.76%)	1,609 (3.58%)	1781 (3.61%)	1,532 (3.05%)	959 (2.67%)	7,568 (3.36%)	34.60 ± 6.22
Growth hormone	Somatropin for injection	940 (2.10%)	975 (2.17%)	1,221 (2.48%)	1,301 (2.59%)	1,584 (4.41%)	6,021 (2.67%)	35.35 ± 5.57
Dopamine agonists	Bromocriptine mesilate tablets	514 (1.15%)	647 (1.44%)	717 (1.45%)	601 (1.20%)	479 (1.33%)	2,958 (1.31%)	31.09 ± 4.89
Gonadotropin releasing hormone analogue	Leuprorelin acetate microspheres for injection	413 (0.92%)	738 (1.64%)	541 (1.10%)	596 (1.19%)	541 (1.51%)	2,829 (1.26%)	33.46 ± 5.21
Gonadotropin releasing hormone analogue	Goserelin acetate sustained-release depot	18 (0.04%)	14 (0.03%)	9 (0.02%)	11 (0.02%)	30 (0.08%)	82 (0.04%)	34.24 ± 5.58
Total		44831	44976	49280	50203	35935	225225	32.99 ± 5.42	

**FIGURE 1:**
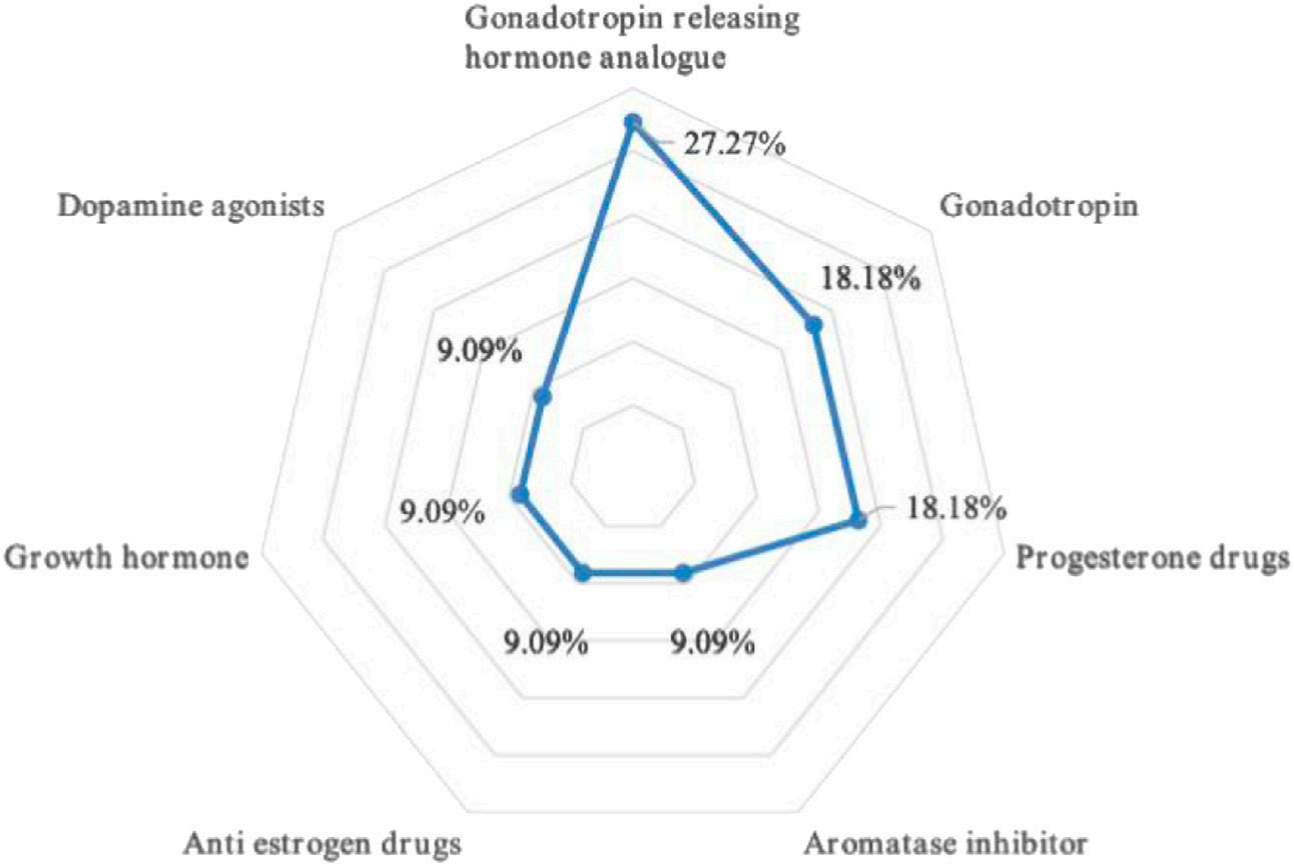
Radar map of drug type distribution.

**FIGURE 2:**
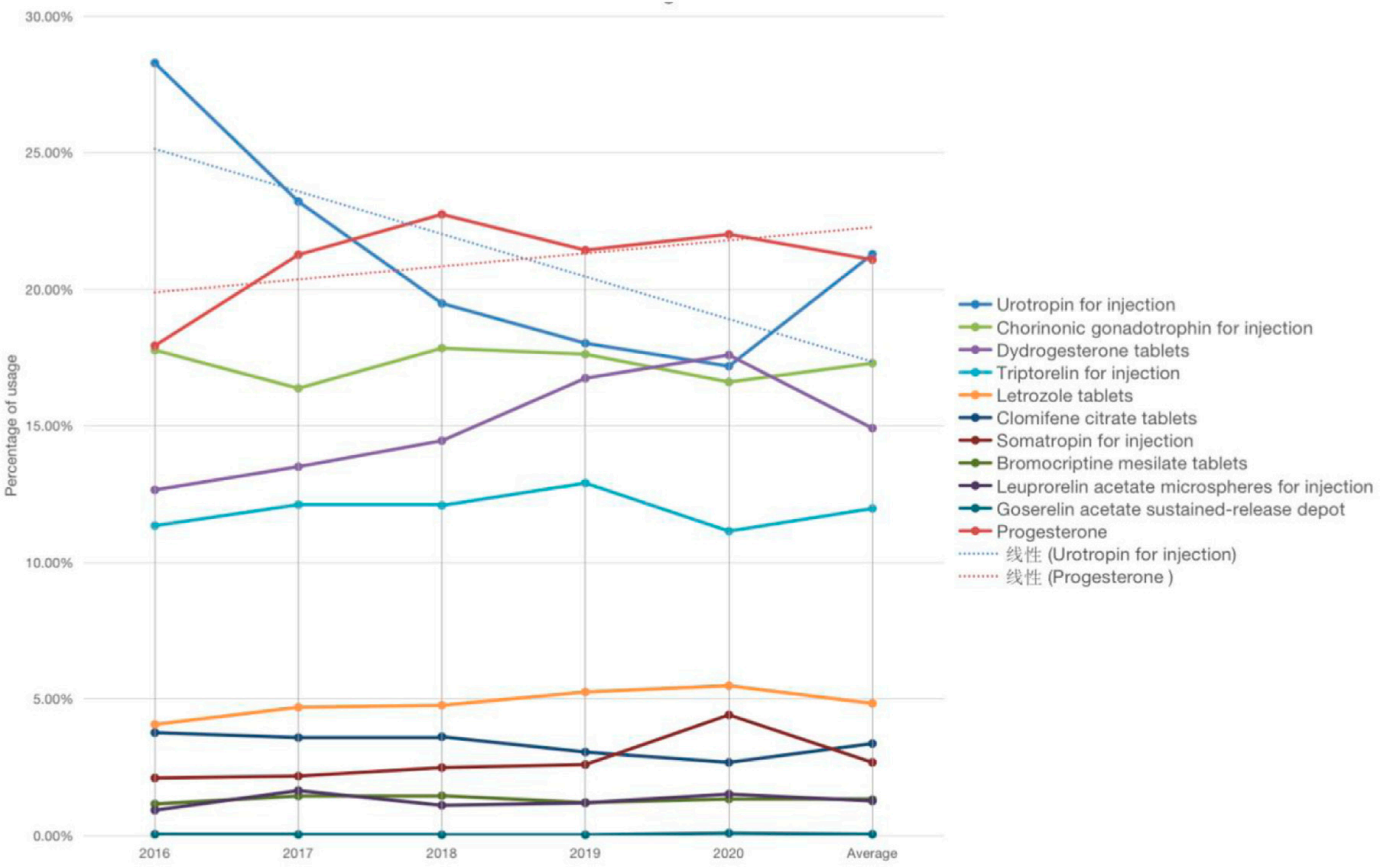
Trends in main therapeutic drugs use.

There are three kinds of gonadotropin releasing hormone analogues, two different types of gonadotropin and progesterone, and one type of aromatase inhibitor, anti-estrogen drugs, growth hormone and dopamine agonist drugs respectively. Refer to Figure 1 for a breakdown of the distribution of corresponding types. The total dosage of urotropin ranked first, followed by progesterone. However, the dosage of urotropin and chorionic gonadotropin decreased during 5 years, with urotropin decreasing the most; the dosage of progesterone and didroxyprogesterone increased, with progesterone increasing the most. Figure 2 shows the corresponding trend change diagram.

### Analysis of drug usage and dosage

When summarizing the data, we found that the drug dosage in the clinical drug treatment scheme for infertility is more flexible and changeable. Although the overall drug selection will be similar, the scheme choice differs in different regions or hospitals. For example, the most common usage and dosage of urotropin for injection is 5,000–10000 units at a time (19598, 50.60%). The second most common is to inject 1,000–3,000 IU each time within 9 days after ovulation or embryo transfer (such as on the 3rd, 6th, and 9th days after ovulation induction) (7,916, accounting for 20.33%). Cashmere gonadotropin for injection is used daily starting (or from the fifth day of the cycle) once 75–150 IU. Seven days later, the dose will be adjusted according to the estrogen level and follicular development, and increased to 150–225 IU per day (20901, accounting for 43.61%). Triptorelin for injection once a day accounted for 91.61%, with 0.1 mg and 3.75 mg being the immense proportions, with 6,956 (25.80%) and 5,989 (22.22%) patients, respectively. Take one tablet of didroxyprogesterone twice a day for habitual abortion with a total of 20,600 (accounting for 61.33%) when used for luteal support in assisted reproductive technology. When treating infertility caused by insufficient endogenous progesterone, one tablet of didroxyprogesterone is given orally three times a day for a total of 3,587 (10.68%) tablets. 675 (accounting for 2.01%) take one tablet orally daily on the 14th–25th days of the menstrual cycle. The use of progesterone was divided into oral, injection and suppository, which were 26239 (55.27%), 17002 (35.81%) and 4,231 (8.91%) respectively.

### Cost

We counted the total and per capita cost of patients using different therapeutic drugs. Triptorelin, urotropin, progesterone, didroxyprogesterone, and leuprorelin were the top five expensive medications. The top five drug expenses per capita for patients receiving complete treatment are goserelin, leuprorelin, triprelin, growth hormone, and didroxyprogesterone. See Table 3 for details.

**TABLE 3:** Cost of therapeutic drugs use per person each year from 2016 to 2020.

Main therapeutic drugs	2016	2017	2018	2019	2020	Amount (RMB)	Number of patients	Per capita (RMB)
Triptorelin for injection	2765277.05	2853807.03	3843686.14	4159085.09	2314620.1	15,936,475.41	26959	591.14
Urotropin for injection	2422586.78	1827378.21	1677661.42	2207593.72	1338171.8	9,473,391.93	47932	197.64
Progesterone	999059.38	1561365.39	2093263.95	2160307.96	1518768.17	8,332,764.85	47472	175.53
Dydrogesterone tablets	1413653.15	1305429.03	1377180.01	1609621.66	1281911.45	6,987,795.30	33591	208.03
Leuprorelin acetate microspheres for injection	672033.2	1002237.2	756399.51	776866.61	675348.8	3,882,885.32	2,829	1,372.53
Somatropin for injection	517735.77	565666.77	724187.21	687106.07	907571.15	3,402,266.97	6,021	565.07
Chorinonic gonadotrophin for injection	236407.31	314520.44	390089.35	391615.3	272688.32	1,605,320.72	38932	41.23
Letrozole tablets	269233.89	269386.84	280316.99	290608.26	192405.95	1,301,951.93	10881	119.65
Bromocriptine mesilate tablets	53639.87	66265.56	67007.5	55573.41	43320.14	285,806.48	2,958	96.62
Goserelin acetate sustained-release depot	35801.51	34194.24	15504.21	33969.92	48450.28	154,309.62	82	1881.82
Clomifene citrate tablets	28537.77	28604.43	30683.6	20359.38	21247.48	143,043.20	7,568	18.90
Total	9413965.68	9828855.14	11255979.89	12392707.38	8614503.64	51,506,011.73	225225	228.69

## Discussion

### Demographic characteristics

According to statistics data, the primary age group of infertility treatment locates between 25 and 34, with an average age of 32.99. There has been no substantial increase in the average age of treatment over the past 5 years, and the change in age is not statistically significant (*p* > 0.05), contrary to the belief of some research that the prevalence of infertility steadily increases with age (Meng, 2014; Sun et al., 2017). The number of patients seeking medical treatment has not increased over the previous 5 years, indicating that the patients’ reproductive demands are mainly concentrated around the age of 33, with the lowest number in 2020. Beyond this age, they give up the focus on breeding offspring, which may be related to the decline of fertility caused by the change in global population structure, environmental pollution and reproductive tract infection. The prevalence of infertility in China appears to be on the rise, although Hu Baoshan and others believe it has remained steady at 25% in recent years (Zhou et al., 2018; Liang et al., 2021), and the number of people treated has not increased. The prevalence of infertility in China looks to be on the rise. However, Hu Baoshan and others believe it has remained stable at 25% in recent years, and the number of individuals receiving treatment has not increased proportionately. Reasons such as the evolution of society, environmental pollution, increased job pressure, delayed marriage and childbearing age, and the number of persons with infertility certificates contribute to a progressive increase in the childbearing age adjustment. Likewise, people’s thoughts are continually evolving and improving.

Guangzhou has the highest proportion of patients. Relevant studies suggest that the infertility prevalence rate of 19,595 first married couples sampled from the population in Guangdong Province from 2002 to 2003 is 14.7% (Hu et al., 2004), which is higher than that of other provinces and cities in China (Zhou et al., 2018; Liang et al., 2021). However, there are other explanations for the varying fraction of infertility treatment patients, including varying diagnostic criteria for infertility, inconsistencies in the precision of calculations, and genuine regional variations in prevalence rates. No conclusion could be drawn from this investigation. Numerous women believe that their age is crucial in determining their fertility. When they reach a particular age, they must resolve any fertility issues. It will become a significant concern if they deviate from this age norm and experience infertility. The rising opportunity cost of women’s motherhood has significantly impeded their childbearing intentions.

### Drug categories

In the statistics of outpatient drugs in key cities in China, the varieties and proportion of injections are the most, including the most common ovulation promotion and urinary stimulation, triptorelin, and progesterone. In addition to oral preparations, injection preparations have become the leading drugs in infertility clinics. Both urinary gonadotropin for injection and chorionic gonadotropin for injection belong to gonadotropin. They are often combined with primary or secondary amenorrhea caused by insufficient secretion of gonadotropin and infertility caused by anovulation; Progesterone is generally used to treat functional disorders caused by progesterone deficiency and is also helpful for pregnancy. Progesterone is used in the most multiple ways; Didroxyprogesterone tablets are used to treat diseases caused by endogenous progesterone deficiency, threatened abortion or habitual abortion caused by progesterone deficiency, and infertility caused by corpus luteum deficiency; Triptorelin for injection is mainly used to reduce the serum concentration of sex steroids to the castration level, such as in hormone-dependent prostate cancer, endometriosis, hysteromyoma, etc.

The statistical material of this study differs slightly from that of earlier research. It has been stated that clomiphene (CC) is the most often utilized medication for inducing ovulation (Zegers-Hochschild et al., 2017). Although clomiphene or gonadotropins are problematic due to ovarian hyperstimulation syndrome and multiple pregnancies, which increase the risk of premature birth, and the rate and expense of related neonatal incidence, they are empirical ovarian stimulation methods, according to numerous sources (Practice Committee of American Society for Reproductive Medicine, 2012; Brown and Farquhar., 2016; Lu, 2019; Bansal et al., 2021). Ragni et al. (2012) compared the randomized controlled non-inferiority test of clomiphene citrate and high-dose gonadotropin *in vitro* fertilization of women with impaired ovarian reserve. It showed that in women with impaired ovarian reserve selected for *in vitro* fertilization, ovarian stimulation with clomiphene citrate or high-dose gonadotropin could lead to similar pregnancy opportunities, but the cost of the former strategy is lower (Kulkarni et al., 2013). In a study that recruited couples with unexplained infertility in a multicenter, randomized trial, Diamond proposed stimulating the ovary with triazole to reduce multiple pregnancies while maintaining a live birth rate compared with gonadotropin or clomiphene citrate (Ragni et al., 2012).

Clomiphene has been the first-line oral therapy for ovarian stimulation in unexplained infertility for over four decades. Clomiphene is a safe and effective oral medicine. However, it is known to cause pervasive side effects, such as anti-estrogen endometrial and anti-cervical mucus, which may diminish the likelihood of conception. Moreover, clomiphene citrate increases the likelihood of multiple pregnancies compared to natural cycles. These drawbacks are primarily attributable to the extended antiestrogenic impact of accumulated clomiphene in the body (the half-life of clomiphene isomer is several days to several weeks) (Sakhavar et al., 2014; Diamond et al., 2015).

There is no direct data about the effect of luteal phase support on fertility enhancement in spontaneous or non-gonadotropin-induced ovulation cycles. Contrariwise, luteal phase deficit is always present in gonadotropin *in vitro* fertilization (IVF) and non-IVF cycles, and progesterone has substantial benefits in reproductive results. Due to its extensive and empirical use from the natural ovulation cycle to assisted reproductive technology, the role of progesterone in promoting the luteal phase remains contentious (Chaudhury et al., 2013; Haas and Ramsey, 2013; Pickar, 2018).

Although gonadotropin or clomiphene citrate is currently considered the first-line program for ovulation induction, our survey results indicate that clomiphene is not physicians’ first choice, which may be related to its efficacy and adverse effects. Although progesterone is not a standard recommendation, the dosage of progesterone in different dosage forms is high due to the conventional thinking mode. There was no significant difference in the overall incidence of congenital malformations among children born to mothers who were naturally conceived or treated with letrozole or clomiphene (Miralpeix et al., 2014). Letrozole is extensively recommended for the treatment of breast cancer in postmenopausal women. Recent evidence indicates that letrozole is an effective ovulation-inducing medication (Sharma et al., 2014). Although the proportion of letrozole in this study is not high, the dosage tends to rise.

### Usage and dosage

Sex hormones are flexible in the treatment of infertility. First-line drugs, especially cashmere gonadotropin and urotropin, are the top five drugs with high dosages. Progesterone oral preparation accounted for the highest proportion, followed by injection and suppository dosage form. Progesterone has variable pharmacokinetic and pharmacodynamic features and relative benefits and merits due to different administration routes (Piette, 2020). A preliminary meta-analysis showed no significant difference in the treatment results of vaginal and intramuscular progesterone (Palomba et al., 2015).

Regarding progesterone dosage, a Cochrane analysis revealed that the standard (90 mg/day) or high (equivalent to or greater than 100 mg/day) vaginal progesterone dose was not diverse in the studies (Zarutskie and Phillips, 2009). Less than 300 mg/day of vaginal progesterone is less effective than 100 mg/day of intramuscular progesterone, however there is no difference in efficacy at 600 mg/day (Yanushpolsky, 2015). Oral administration constitutes the most significant proportion in this study, which may be attributable to its convenience. Currently, most scientific information is derived from meta-analyses of observational research and a few randomized controlled trials. Future considerations should include implementing randomized trials and examining the promising efficacy of high-dose subcutaneous progesterone. Gonadotropin-releasing hormone (GnRH) agonists should be utilized to initiate a fresh cycle of multiple ovulation during controlled ovarian hyperstimulation (COH). There is a scientific disagreement about progesterone administration scheme, particularly the route, dose, and timing of administration, as well as the potential interactions with other medicines, which require additional investigation.

### Drug amount

The highest amount of drugs used for ovulation induction in IVF clinic is triptorelin, urotropin and progesterone. The top per capita costs are goserelin, leuprorelin, triptorelin and growth hormone. GnRHa drugs have the highest per capita costs. Most ovulation-inducing medications and GnRHa sex hormone therapies, which are utilized for treatment prior to ovulation induction, are among the top-selling pharmaceuticals. Progesterone injection is an essential national drug. Compared with other progesterone preparations, the price is very low, but the use frequency is also very high. The amount of outpatient medication in IVF is obviously out of sync with DDDs. Because IVF is high-tech, the ovulation inducing drugs used are relatively new and the price is relatively high. There is a certain tendency of drug use in the research data of different years. The change of amount in different years and the drug consumption structure show that the drug use in hospitals in different regions is consistent. It shows that everyone generally agrees with the choice of main therapeutic drugs.

## Conclusion

Our investigation tallied the prescriptions written by outpatients for reproductive aid from January 2016 to December 2020. According to the statistics, there was no substantial increase in the average age of patients over the past 5 years. However, the opportunity cost of female fertility increased, Severely impacting fertility intentions. The selection of medications is reasonable overall. In this study, the dosages of urotropin and chorionic gonadotropin advised by the guidelines are likewise excessive. However, it should be mentioned that clomiphene has traditionally been the first-line oral medication for ovarian stimulation in cases of unexplained infertility. However, our statistical data indicate that the dosage of progesterone is high. In addition, certain medications will increase the financial burden on patients. Future research will focus on enhancing the degree of rational drug use among outpatients and realizing the economical, safe, and effective use of pharmaceuticals to lessen the economic burden on patients.

## Data Availability

The original contributions presented in the study are included in the article/Supplementary Material, further inquiries can be directed to the corresponding author.
