# Supplementary material for: Real-world prescription patterns for reproductive assistance patients in China: A trend analysis from 2016 to 2020
Source: Front Pharmacol. 2022 Nov 17;13:1021150. doi: 10.3389/fphar.2022.1021150 (PMC9713242; doi:10.3389/fphar.2022.1021150)

**Accordance and Informed Consent**

This study was approved by the hospital prescription analysis cooperation project supervised by the China Medical Association. The requirement for informed consent from the participants was not provided because the data were analysed anonymously.

The accordance and informed consents are in the hospital information system of the hospital public computer. Every participant signed a statement that “The patient agreed that the hospital would retain my information for scientific research” in the form of electronic signatures. Patient’s privacy and confidentiality of data were secured by the principle investigator. The following picture is one of the accordance and informed consents.


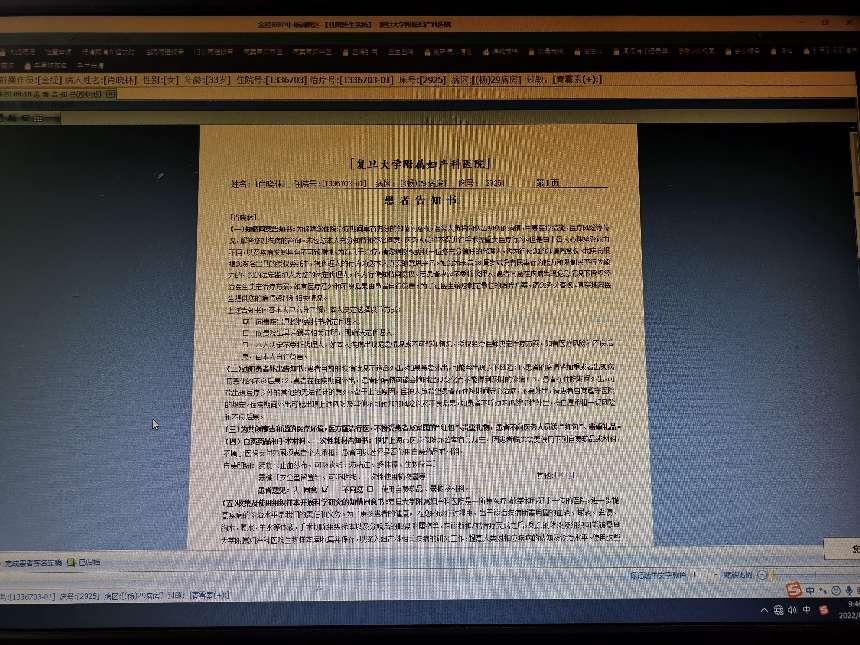


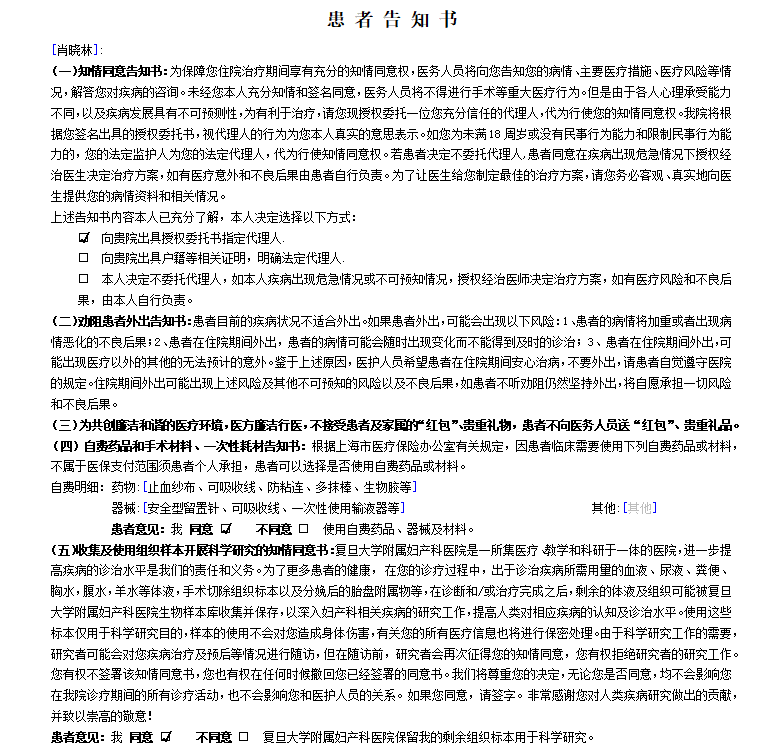


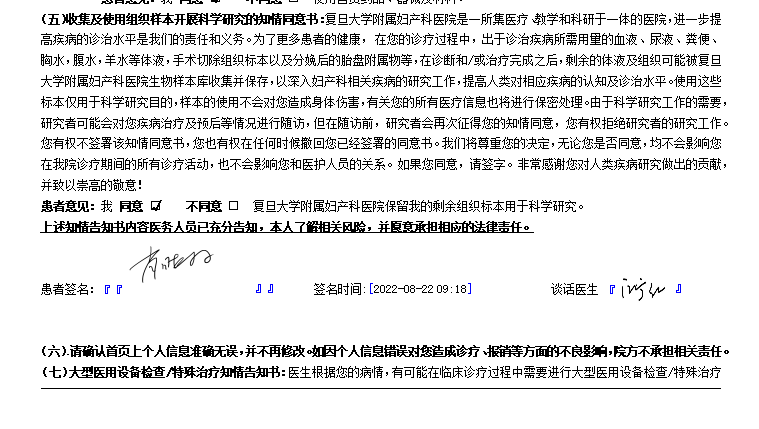

Supplement: Supplementary file 1 [file Table1.DOCX]
